# Osteocyte gene expression analysis in mouse bone: optimization of a laser-assisted microdissection protocol

**DOI:** 10.1093/jbmrpl/ziae078

**Published:** 2024-06-24

**Authors:** Mathilde Palmier, Marlène Maître, Hélène Doat, Thierry Lesté-Lasserre, Delphine B Maurel, Claudine Boiziau

**Affiliations:** Inserm, University of Bordeaux, BioTis Laboratory UMR 1026, Bordeaux, France; Inserm, University of Bordeaux, Neurocentre Magendie UMR 1215, Bordeaux, France; Inserm, University of Bordeaux, Neurocentre Magendie UMR 1215, Bordeaux, France; Inserm, University of Bordeaux, Neurocentre Magendie UMR 1215, Bordeaux, France; Inserm, University of Bordeaux, BioTis Laboratory UMR 1026, Bordeaux, France; Inserm, University of Bordeaux, BioTis Laboratory UMR 1026, Bordeaux, France

**Keywords:** cortical bone, bone cells, gene expression, UV laser

## Abstract

Among bone cells, osteocytes are the most abundant, but also the most challenging to study because they are located inside a dense mineralized matrix. Due to their involvement in bone homeostasis, diverse tools are needed to understand their roles in bone physiology and pathology. This work was aimed at establishing a laser-assisted microdissection protocol to isolate osteocytes and analyze their gene expressions. The goal was to overcome the limitations of the technique currently most used: RNA extraction from the whole bone. To perform laser microdissection and subsequent gene expression analysis, the five main steps of the protocol have been adapted for the bone tissue. After testing many parameters, we found that the best options were (1) take unfixed snap-frozen tissue, (2) cryosection with a supported tape system to improve the tissue morphology if necessary, (3) microdissect regions of interest, and (4) recover the bone pieces by catapulting, if feasible, or by gravity. Finally, RNA extraction (5) was the most efficient with a precipitation method and allowed quantifying the expression of well described osteocyte genes (*Gja1/Cx43*, *Phex*, *Pdpn*, *Dmp1*, *Sost*). This work describes two protocols optimized for femur and calvaria and gives an overview of the many optimization options that one could try when facing difficulties with laser microdissection.

## Graphical Abstract

**Graphical Abstract:**
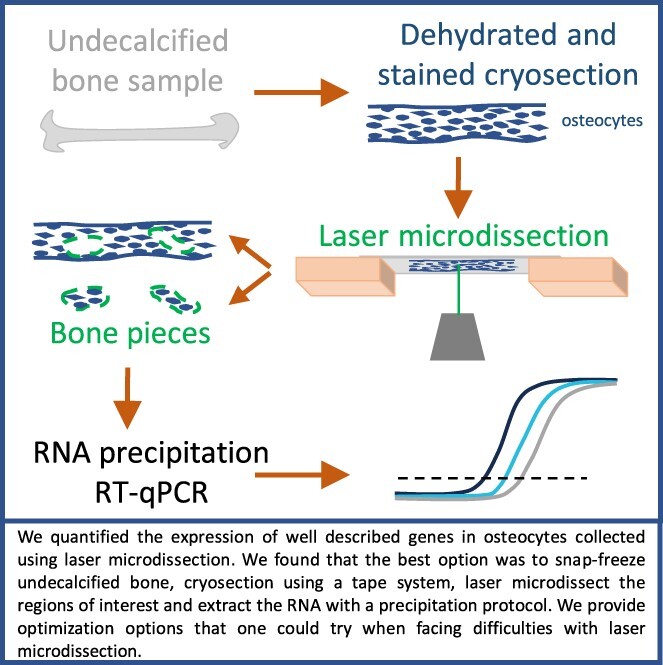

## Introduction

Bone is composed of a compact, mineralized matrix and is a dynamic organ able to remodel and repair. Thus, it maintains its functions of body weight support and protection for vital organs and regulates the calcium and phosphate homeostasis. Three main cell types sustain bone activity, namely osteoclasts, osteoblasts, and osteocytes. Osteoclasts resorb the matrix where the bone needs to be renewed and osteoblasts form the matrix. Osteocytes are terminally differentiated osteoblasts that become embedded into their own matrix to form a network.^1^ Among bone cells, osteocytes are the most abundant, but also the most challenging to study because of their location inside the hard matrix. Nevertheless, significant advancements were made over the past decades showing that osteocytes have critical roles in bone homeostasis. This is due to their ability to receive mechanical and hormonal signals and to communicate with surrounding and distant cells.^2^ These findings result from the development of *in vitro* and *in vivo* tools, mainly in mice, to study osteocyte biology. *In vitro* immortalized cell lines such as MLO-Y4, Ocy454, or IDG-SW3 have been essential for the investigation of osteocyte differentiation and mechanosensing.^3^^,^^4^ Although these *in vitro* models are necessary for the comprehension of osteocyte physiology, they are complementary to *in vivo* ones. Transgenic *Dmp1*-Cre and *Sost*-Cre animal models targeting osteocytes enabled the study of gap junctions and communication with osteoclasts.^5^ However, some limitations such as off-target Cre recombination make them imperfect. In addition to these elaborated techniques, RNA extraction for subsequent gene expression analysis is an efficient way to analyze cell biology. For long bones, the periosteum is scrapped, the bone marrow is flushed, and digestion/decalcification steps are applied to remove cells from the surface. Even if effort is made to remove contaminations with other cells, structures embedded in the matrix remain present (blood vessels). It is even harder to obtain RNA from osteocytes from the femoral head, the vertebrae or the calvaria, where bone marrow cannot be removed efficiently. This results in the best case in the analysis of an osteocyte-enriched content.^6^ Moreover, whole bone RNA extraction does not allow to discriminate between osteocyte populations based on their location in bone or on their differentiation stages.

Laser-assisted microdissection, also called LCM (Laser Capture Microdissection) or LMD (Laser Microdissection), was developed to allow the isolation of specific cells in tissue sections under the control of a microscope. This tool was initially used for soft tissue dissection, such as tumor or brain,^7^ and was more seldomly described for bone. Different technologies and protocols were published, but very few articles mentioned results obtained after the use of this technique.^8–10^ The main challenge is to ablate the matrix with the laser to collect enough cells and obtain RNA to analyze. LCM needs to be especially customized for the working conditions and the studied tissue in order to be successful. Many steps are required in the procedure and each of them must be optimized: sample preparation, histological section, dehydration and staining, laser microdissection, tissue lysis, and RNA extraction. The objective of this work was to specifically isolate osteocytes and analyze the expression of some of their genes known as markers. We adapted laser microdissection protocols found in the literature to our working conditions based on three critical parameters, the ability to cut bone with the laser, the RIN (RNA Integrity Number) value after each step, and the RT-qPCR threshold cycle values (Ct). The present article describes the optimized method for two mouse bone types, femur and calvaria, and gives an overview of the many optimization options that one could try for each step when facing difficulties with an LCM procedure.

## Materials and methods

Step-by-step protocols and product information are available in file S1.

### Bone isolation and preparation

All mouse experimentations were done in accordance with the European guidelines in the animal facility of the University of Bordeaux (agreement #A33-063-917). Supernumerary male and female adult C57BL6/J mice (3-7 months) were euthanized by cervical dislocation; femurs from both hind limbs and/or calvaria were immediately isolated. We tested four different sample preparations found in the literature (one animal per sample preparation method): (A) decalcification with conventional ethylene-diamine-tetraacetic acid (EDTA), where each bone was immersed in 10 mL of a solution of 10% (w:v) EDTA in RNA*later*™ (Invitrogen, Fisher Scientific, Illkirch, France) at pH 5.2, and gently agitated for 72 h at 4 °C^11^; (B) decalcification within 20 mL of 20% (w:v) EDTA for 2 days at 4 °C using constant agitation followed by fixation for 1 h at 4 °C with Methacarn (methanol 60%, chloroform 30%, and glacial acetic acid 10%), and dehydration for 1 h in ethanol 100% at 4 °C^9^; (C) direct snap-freezing (without isopentane) without any pre-treatment or decalcification^8^^,^^10^^,^^12^; (D) no decalcification, and immersion for 24 h at 4 °C in 10 mL of RNA*later*™.^13^ All the bones were embedded into OCT, Optimum Cutting Temperature gel (TFM-C, MM France, Brignais, France or SCEM, SECTION-LAB Co. Ltd., Yokohama, Japan) without prior rinsing step, and snap-frozen in a cryomold (Methods A–C) or frozen at −26 °C^13^ (Method D). The samples were stored at −80 °C before being sectioned using a cryostat.

### Cryosections

A Leica CM 3050S cryostat (Leica Microsystems, Wetzlar, Germany) was used for cryosectioning the tissue blocks. Blades specifically designed for hard tissue were used (MM35P, MM France, Brignais, France). We tested the section thicknesses of 5–10 μm for undecalcified bone or 8–16 μm for decalcified bone. Two to three sections were then mounted onto PEN MembraneSlides or PET FrameSlides (UV technology, Carl Zeiss, Jena, Germany). MembraneSlides are glass slides covered with a PEN (polyethylene naphthalate) film and FrameSlides are PET (polyethylene terephthalate) films attached to a metal frame. To improve the section quality, either the Kawamoto LMD film method (SECTION-LAB Co. Ltd.) or the CryoJane Tape-Transfer System (Leica Microsystems, Wetzlar, Germany) were also tested according to published protocols.^14^^,^^15^ Briefly, in the CryoJane system, the microdissection Membrane/FrameSlides were coated with an adhesive solution. An adhesive tape was then placed onto the tissue block fixed to the sample holder inside the cryostat. The frozen section stayed attached to the tape while being cut. The tape was placed onto a MembraneSlide or FrameSlide with the frozen tissue facing the adhesive coating. A UV flash polymerized the adhesive coating to trap the section and the tape was peeled off to transfer the tissue section from the tape to the slide (2–3 sections per slide). For the Kawamoto method, the tape was also placed onto the tissue block to obtain a section adhering to the tape. Then, the tape was fixed onto a frame provided by the manufacturer, and the tissue was directly microdissected together with the tape (at least two slides were prepared for each analyzed condition). All the steps were performed at −27 °C ± 1 °C.

### Section treatment: dehydration and staining

Cell nuclei were stained with Cresyl Violet (Sigma-Aldrich Co., St. Louis, MO, USA) according to the following procedure: each slide was dived into baths of decreasing grades of ethanol (95%, 75%, and 50%) for 40 s, stained with Cresyl Violet 1% in 50% ethanol (10 s), and dehydrated using increasing grades of ethanol (50%, 75%, 95%, 100% twice) for 40 s each. Then, the slides were air dried and left on the bench at room temperature or processed through the LCM procedure. One should note that when using the Kawamoto LMD film, the three ice-cold ethanol baths with decreasing concentrations before staining were skipped and the time of the dehydration steps was reduced to 30 s to keep the tape adherent to the frame.

### Laser-assisted microdissection

Sections were microdissected using a 355-nm UV laser, coupled with an inverted microscope and software (P.A.L.M MicroBeam, Carl Zeiss, Jena, Germany). A 20× magnification was used and laser energy and focus settings were adapted according to the microdissected samples. Nevertheless, in most of the cases, bone ablation was performed at 78% of the total energy, with a focus of 75%, and a speed varying from 10% to 30% depending on the section quality. One cycle corresponded to one passage of the laser. The laser impulse was set at 80% of the total energy with a focus of 70%. The area collected depended on the duration of the laser microdissection step. Bone pieces were stored in lysis reagent in a microtube (see “Tissue lysis, RNA extraction and RIN determination”) at −80 °C until RNA extraction. Two collection methods of the bone pieces were tested, either by gravity with the addition of a coverslip (0.13-0.16 mm, Fisher Scientific) immobilized under a FrameSlide or by lifting the piece—with a laser impulse—into an AdhesiveCap (500 μL, Carl Zeiss) placed above the bone section. One should note that when using the Kawamoto method, the option “joint cut” was selected to prevent the bone piece from falling and the laser impulse was applied to the joint between the bone piece and the rest of the section to catapult it into the cap placed above.

### Tissue lysis, RNA extraction, and RIN determination

Samples (four sections for each condition) were subjected to different kinds of lysis before the RNA isolation step: (1) RLT buffer (Qiagen, Hilden, Germany) containing 1% (v/v) of β-Mercaptoethanol (Merck, Darmstadt, Germany) (“RLT/βME solution”); (2) RLT/βME solution and 2 rounds of 30 s of bead-beating at 5000 rpm, with 12 zirconia-silica beads (0.1-mm diameter, BioSpec Products, Bartlesville, OK, USA), using a Precellys® homogenizer (Bertin Technologies, Aix en Provence, France); (3) RLT/βME solution and 30 s of ultrasonication “Force 1” (Digital Sonifier model S-150D ultrasonic cell disruptor, Branson, Teltow, Germany) in ice to avoid heat; (4) lysis solution composed of TRI reagent® (Molecular research center, Inc., Cincinnati, OH, USA). RNA was extracted from bone pieces either with columns of the RNeasy® Micro Kit (Qiagen)^16^ or by precipitation; two precipitation methods were assessed: either TRI reagent®**/**chloroform method or MasterPure™ Complete DNA & RNA Purification Kit (Lucigen, Teddington, UK). Samples were stored at −80 °C and run on an Agilent Bioanalyzer RNA Pico Chip (Agilent Technologies, Santa Clara, CA, USA) to determine the total RNA Integrity Number (RIN). RNA concentration was assessed, but it was too low to be quantified by commonly used methods such as spectrometry with a NanoDrop™ spectrophotometer.

### RT-qPCR

RNA was processed and analyzed according to the MIQE guidelines.^17^ Briefly, cDNA was synthesized from total RNA by using qScript XLT cDNA SuperMix (Quanta Biosciences, Beverly, MA, USA). One-tenth of the cDNA was retained for nested pre-amplification PCR. Direct qPCR reactions were performed with a LightCycler® 480 Real-Time PCR System (Roche Diagnostics, Meylan, France) and primers were designed to span an exon–exon junction (Table 1). qPCR reactions were done in duplicate for each sample by using LightCycler 480 SYBR Green I Master (Roche Diagnostics) in a final volume of 10 μL. An initial denaturation step at 95 °C for 5 min was followed by 45 cycles of 95 °C for 15 s and 61 °C for 30 s. Nested pre-amplification PCR was also performed on the LightCycler® 480 (Roche Diagnostics) using PerfeCTa PreAmp SuperMix (Quanta Biosciences) with template cDNA and primers at 0.5 μM. The reaction mixture was subjected to 10 PCR cycles consisting of an initial denaturation step at 95 °C for 2 min followed by 10 cycles of 95 °C for 10 s and 60 °C for 3 min. Unincorporated primers were removed by exonuclease (NEB, Ipswich, MA, USA) digestion. The second amplification was performed using LightCycler® 480 SYBR Green I Master (Roche Diagnostics) on amplified cDNA in a reaction of 10 μL, using the same primers at 0.6 μM. An initial denaturation step at 95 °C for 5 min was followed by 45 cycles of 95 °C for 15 s and 61 °C for 30 s. To confirm the specificity of DNA amplifications, PCR products were run in an automated electrophoretic DNA separation system (Labchip GX II, Caliper life sciences, MA, USA); their size was as expected. For each primer pair, a no-template-control without cDNA was also tested, and no PCR products were observed.

**Table 1:** Primer sequences.

**Genes**	**GenBank ID**	**Forward primers (5′-3′)**	**Reverse primers (5′-3′)**
*Eef1a1*	NM_010106	TGTTGATATGGTCCCTGGCAA	CTTTGATGACACCCACAGCAA
*Gapdh*	NM_008084	CGATGCCGGGGCTGGCATT	TGGGTGGTCCAGGGTTTCTT
*Sdha*	NM_023281	TACAAAGTGCGGGTCGATGA	TGTTCCCCAAACGGCTTCT
*Gja1*	NM_010288	ACTCTCCTTTTCCTTTGACTTC	TGGACCTTGTCCAGCAGCTT
*Pdpn*	NM_010329	CGTCTTGTTAGCCATTGGCTT	GGCAAGTTGGAAGCTCTCTTA
*Dmp1*	NM_001359013	TGAAGAGAGGACGGGTGATTT	CCCAGCTCCTCTCCAGATT
*Phex*	NM_011077	CAATTCCTATAGACCAGAAGCT	AGTGGAATTTCGTGGACAGTTA
*Sost*	NM_024449	TCCTGAGAACAACCAGACCAT	GCGGCATGGGCCGTCTGT

The qPCR data were exported and analyzed in a software (Gene Expression Analysis Software Environment) developed at the Neurocentre Magendie (Bordeaux, France).

## Results and discussion

**Figure 1:**
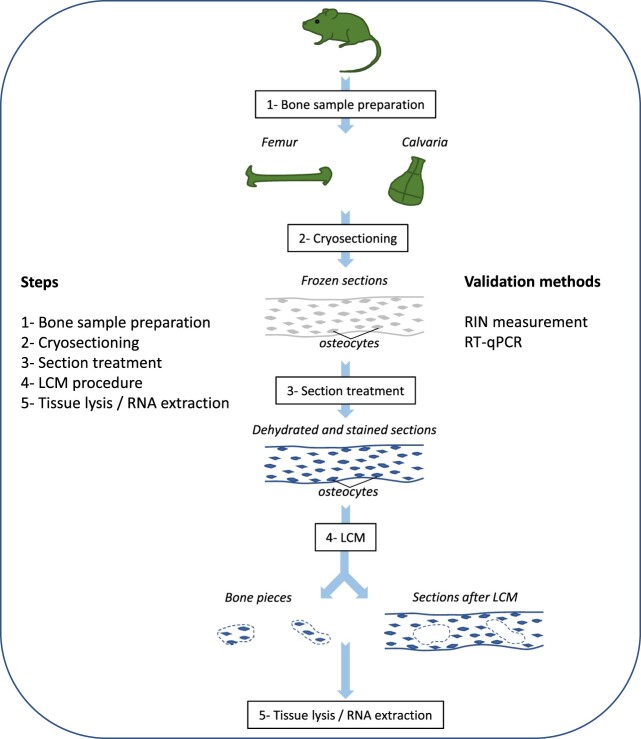
Successive steps to isolate mRNA and analyze gene expression from osteocytes. The article describes the five main steps optimized to be able to perform RT-qPCR.

Our first attempts of LCM with mouse bones were based on a protocol commonly used for rat brain studies^18^ but remained unsuccessful due to the difficulty to cut bone tissue with the laser beam. Therefore, an optimization of each step of the protocol was necessary to apply LCM to bone and to ensure the preservation of RNA: (1) bone sample preparation, (2) cryosection, (3) treatment of the sections (staining and dehydration), (4) laser microdissection and sample collection, and (5) tissue lysis and RNA isolation. After each step, RNA quality was assessed based on the RIN. Finally, RT-qPCR was performed to validate that we successfully collected and studied osteocytes (Figure 1).

### Bone sample preparation

Working with frozen sections gives better chances to recover good RNA quality.^19^ However, preservation of the tissue morphology can be challenging.

We studied bones from mature animals with well mineralized bones (3- to 7-mo-old mice). When they are used for histology or imaging, it is common to perform a decalcification step for several days to improve the sectioning performances. For LCM and gene expression analysis, the decalcification step is likely to easily degrade RNA.^11^

The aim of this step (Step 1) was to select a freezing method enabling the cutting of the bone samples with a cryostat, and to obtain a good RNA quality (RIN > 7.0). After animal euthanasia and dissection, four different bone preparations (see “Bone isolation and preparation”) based on published methods^8–13^ were tested to evaluate the impact of decalcification (Methods A and B) or snap-freezing (Methods C and D) on RNA quality, as well as the benefit of RNA*later*™ impregnation (Methods A and D). EDTA is a calcium chelating agent commonly used for bone decalcification, and RNA*later*™ is an aqueous, nontoxic reagent used to protect the integrity of RNA in unfrozen tissue samples. Methacarn was tested in Method B after decalcification; it is a fixative reagent previously described in paraffin embedding protocols for RNA preservation.^9^^,^^20^

High RIN values (>8.0) were obtained except for the method using Methacarn (Table 2, step 1). Methods A and B, including the decalcification step, gave, respectively, the highest and the lowest RIN values. It validated the use of RNA*later*™ in an acidic solution during decalcification. However, fixation with Methacarn was deleterious. Among the methods without decalcification, RNA*later*™ used in Method D before snap-freezing did not improve the RIN value compared with Method C.

**Table 2:** Validation of the successive steps by assessment of the RNA integrity (RIN).

Sample preparation method	Specificity of the procedure	RIN
**Step 1 (bone preparation)** ^a^		
A	Decalcification in 10% EDTA- RNA*later*™*,* pH 5.2+ snap-freezing	9.6
B	Decalcification in 20% EDTA-Methacarn + snap-freezing	6.5
C	No decalcification, snap-freezing	8.7
D	No decalcification, incubation in RNA*later*™ + freezing	8.3
**Step 2 (cryosection)** ^b^		
A	Standard cryosectioning, FrameSlide	6.1, 7.8, 9.6
C	Standard cryosectioning, FrameSlide	5.3, 6.8, 8.7
**Step 3 (section treatment)** ^c^		
A	Standard cryosectioning + dehydration + staining +45 min/2 h	7.9/8.0
**Step 4 (LCM procedure)** ^d^		
A	Bone pieces collected with LCM	nd
	Section remaining after LCM	5.6, 7.5
C	Bone pieces collected with LCM	nd
	Section remaining after LCM	3.4, 5.8
**Step 5 (tissue lysis)** ^e^		
A	RNeasy® Micro Kit (bone pieces)	nd^f^
	RNeasy® Micro Kit (section remaining after LCM)	5.6, 7.5^f^
A	Precellys® + RNeasy® Micro Kit (bone pieces)	nd
	Precellys® + RNeasy® Micro Kit (cryosections)	7.0
A	Sonication + RNeasy® Micro Kit (bone pieces)	nd
	Sonication + RNeasy® Micro Kit (cryosections)	7.0
A	TRIspin method (bone pieces)	nd
	TRIspin method (cryosections)	7.3

a Validation of step 1: after the different methods (A, B, C, D) were used to prepare the samples, four 10-μm-thick tissue sections were performed for each bone sample, and lysed all together in RLT/βME solution, before RNA extraction with the RNeasy® Micro Kit.

b Thicknesses 5–10 μm. One RIN value per animal, results for three animals.

c Validation of step 3: after sample preparation A, tissue sectioning, dehydration and staining were performed; sections were left on the bench at room temperature; after 45 min or 2 h, they were lysed in RLT/βME solution, before RNA extraction with the RNeasy® Micro Kit. This experiment was performed one time, with one animal.

d Validation of step 4: after sample preparation A and C, sections after LCM (45 min, 1.0 ± 0.3 mm^2^) were lysed in RLT/βME solution, followed by RNA extraction with the RNeasy® Micro Kit. One RIN value per animal, results for two animals. nd: not detected.

e Validation of step 5: after sample preparation A, either frozen sections (after step 2) or bone pieces (after 2 h of LCM) were chemically and/or mechanically lysed before RNA extraction. nd: not detected. This experiment was performed one time, with one animal.

f Data from the validation of step 4.

Among the three methods giving the best RIN values, we selected Methods A and C to perform the next steps.

### Cryosectioning

Good-quality bone cryosections are crucial for the laser to cut efficiently (Step 2). They determine the success of the LCM step. Three parameters are of great importance: the section thickness, morphology, and flatness.

In the LCM procedure recommended by the manufacturer, cryosections are collected onto a specific slide covered with a polymer film (PEN or PET) on one side. It maintains the shape of the bone piece when it is catapulted by a single laser impulse into an adhesive cap. Thus, the laser cuts the film and the tissue section simultaneously. We tested different slides obtained from the manufacturer: MembraneSlides, glass slides covered with a PEN film, and FrameSlides made of a PET film mounted onto a metal frame. In both cases, the laser cut more efficiently (with less cycle repetitions for the same percentage of energy) when reaching the bone section first, meaning that the slide had to be placed in the device with the tissue facing the objective. Consequently, it was impossible to use MembraneSlides, and only FrameSlides were used in the following steps.

Even after decalcification, the bone collagen matrix is dense, and once collected on a slide, the sections are not always flat. These are the reasons why the laser hardly ablates the bone matrix. We tested different section thicknesses, with 5 and 8 μm being optimal for, respectively, decalcified (Method A) and non-decalcified bone (Method C). This is comparable to what is done for bone and cartilage in general,^9^^,^^13^^,^^21^ especially for undecalcified bone.^22^ The drawback is that the thinner the section, the lower the number of osteocytes collected.

Bone cryosectioning is especially challenging when performing thin sections (10 μm and less) of undecalcified bone. In order to obtain flat sections with an intact morphology, we tested two tape-based sectioning methods: Kawamoto LMD film^14^ and CryoJane method.^15^ We compared both methods with standard cryosectioning: direct transfer of the frozen section onto the FrameSlide.

The CryoJane Tape-Transfer System requires several steps and an additional coating solution to transfer the tissue section from the tape to the slide. This gives good results in terms of morphology preservation, but we obtained degraded RNA compared with the standard cryosectioning (not shown). In contrast, others used CryoJane Tape-Transfer System without any deleterious effect on the mRNA quality and analysis.^8^^,^^13^^,^^15^

The Kawamoto LMD film method was introduced in 2012 and then updated in 2020.^14^^,^^23^ This method was developed in combination with a Leica LMD 7400 using gravity to collect the microdissected bone pieces below the tape and frame. We found this method fast and efficient to maintain bone morphology. We combined it with our LCM method using laser impulse to collect the microdissected bone piece in the adhesive cap above the tape and frame.

The method assessment was based on the preservation of the tissue morphology, reproducibility of section quality, the duration, and the complexity of the procedure. Both methods using a tape resulted in a better preservation of the morphology and reproducibility than the standard cryosectioning. The Kawamoto LMD film had the best performances and was a quicker and easier procedure.

### Dehydration and staining of sections

Dehydration is performed for two reasons: to get thin sections cut efficiently by the laser beam and to limit RNase activity during the microdissection process. Staining the sections before LCM helps to better identify tissue structures and cell nuclei. We used Cresyl Violet as it gave good results in other tissues such as brain and mammary glands.^18^^,^^24^ Cresyl Violet is dissolved in ethanol and binds to negatively charged nucleic acids. Unlike Hematoxylin–Eosin staining, which is also used in this context, it avoids re-hydration in water.^8^^,^^21^

Each slide was dived into baths of decreasing grades of ethanol for 40 s, stained with Cresyl Violet, and dehydrated using increasing grades of ethanol for 40 s each. When using the Kawamoto LMD film, the dehydration and staining protocol was adapted to keep the tape adherent to the frame (see “Section treatment: dehydration and staining”).

To evaluate the effectiveness of dehydration on RNA preservation, we measured the RIN value of stained sections (after step 3) left on the bench at room temperature, and air-dried for either 45 min or 2 h. We obtained RIN values of 7.9-8.0. As these values were in the range of values obtained after step 2, we concluded that the dehydration and staining steps were not deleterious and may limit the RNA degradation over time. We also found no difference between the RIN when the slides were left 2 h or 45 min on the bench (Table 2, step 3).

### Microdissection and collection of osteocyte-containing bone pieces

Optimization of the previous steps allowed efficient cutting with the laser. As explained in “Cryosectioning”, the laser microdissection was more efficient when the tissue faced the objective (face down), to make the laser cut bone first. Despite that, it is important to note that depending on the area in the section and on the ability to fine-tune the focus, more than one cycle was necessary to completely ablate bone.

**Figure 2:**
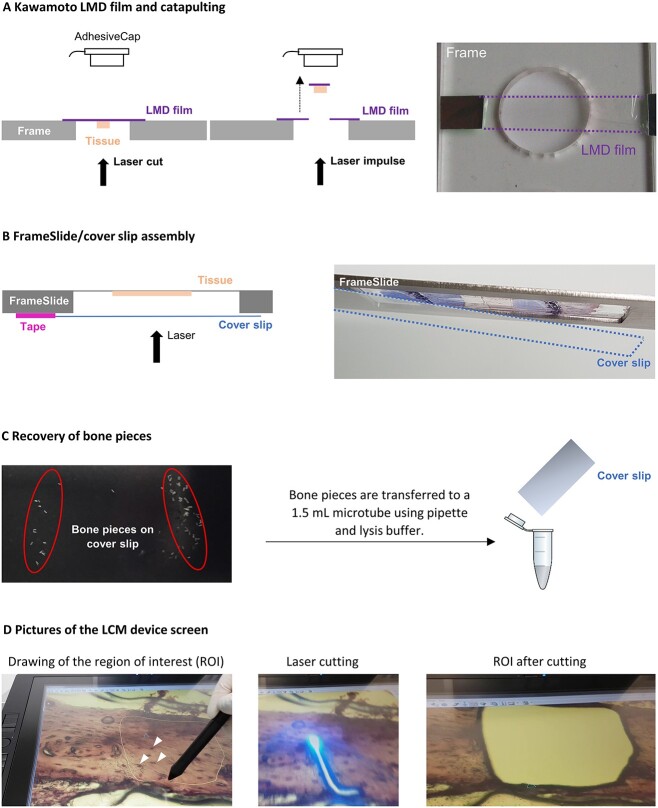
Two different strategies to collect dissected bone pieces. Either the tissue was microdissected from the Kawamoto LMD film and catapulted, or a cover slip was immobilized below the FrameSlide to collect the microdissected pieces by gravity. (A) Scheme of the combination of Kawamoto LMD film and catapulting, and example of a piece of tape onto the frame, (B) scheme and example of the FrameSlide/cover slip assembly, (C) picture of the bone pieces on the cover slip on a dark background and scheme of bone pieces recovery method, and (D) pictures of the screen showing the bone section under the microscope before (drawing of the ROI), during and after the laser cutting, arrowheads show osteocyte nuclei stained with Cresyl violet.

Additionally, depending on the size of the bone pieces, their collection methods had to be adapted. Due to the calvaria bone structure, the bone pieces microdissected around bone marrow cavities were smaller than bone pieces from the femur. Thus, we could lift them by laser pressure catapulting and collect them in the adhesive cap placed above (Figure 2A). Most of the time, with larger pieces from the femur, we could not find any adequate laser impulse parameters and they mostly fell onto the objectives just below. The Zeiss device does not offer the possibility to collect through gravity, unlike the Leica device. To overcome this difficulty, we created an assembly of a FrameSlide and a cover slip (Figure 2B). The tissue sample was placed facing the laser so that it was between the PET membrane and the cover slip. Thanks to the metal frame, there was a space between the tissue and the cover slip so the pieces could fall. Once on the cover slip and after 2 h of microdissection, the bone pieces were transferred into a microtube using the lysis buffer (Figure 2C). This last step was particularly critical and could lead to the loss of some bone pieces falling next to the tube.

The Zeiss method is advertised to be a “noncontact method,” avoiding contamination with other cells. However, using our adapted technique, some residues of tissue surrounding the areas of interest could fall with the bone pieces if they were not strongly attached to the FrameSlide. In this case, a combination of the Kawamoto method with a cover slip below could be useful.

Different technologies of laser microdissection are available on the market. UV and a combination of both UV and IR technologies were used by others for bone and cartilage. Among the publications that mentioned the RIN, a combination of UV and IR was often used.^12^^,^^22^^,^^25^^,^^26^ In these experiments, the RIN values after LCM ranged from 2.7 to 8. Regarding the UV only technique, Rottgers et al.^21^ and Marek et al.^27^ reported values from 2.9 to 8.6. In many other publications, the RIN value was not mentioned, presumably because the quantity recovered was too low to be analyzed as it was reported with other tissues or cells.^24^^,^^28^

To evaluate the impact of the laser microdissection on the RNA quality, we first attempted to measure the RIN from the collected bone pieces, but the Bioanalyzer could not detect any material. It is admitted that when the RIN is impossible to measure from tissue pieces, it can be considered as similar to the RIN from the remaining section after LCM.^24^ Thus, we calculated the RIN difference between cryosections (Step 2) and the rest of the section on the PET membrane after LCM. The RIN was decreased by about 2 after 2 h of LCM (Table 2, step 4). The UV system was designed to minimize its impact on microdissected samples as mentioned by Vandewoestyne et al.^28^ Indeed, there is minimal heat transfer from the part where the laser is focused to adjacent parts, and the nonfocused laser scattered light should not be deleterious. However, Nelson et al.^29^ mentioned that damage can still occur, even if the wavelength of the UV laser is chosen to avoid the nucleic acid absorbance peak. They also specified that the damage caused should be greater for small areas, such as single cells, than on larger multicellular areas. In our case, we minimized the laser impact on mRNA by collecting bone parts containing multiple osteocytes rather than collecting one osteocyte at a time (Figure 2D).

The choice of the duration of the LCM step after staining must rely both on the tissue endogenous RNase activity, and on the number of cells collected during this time. Within 2 h, we could collect 2.5 ± 0.1 mm^2^ of femur and 1.5 ± 0.1 mm^2^ of calvaria, corresponding to about 1500 and 350 osteocytes, respectively, for a section thickness of 5 μm. This is in accordance with the number of cells collected reported in the literature, varying from 10 to 1200.^9^^,^^13^^,^^30^ For calvaria, the bone area recovered was smaller than for femur. This is due to the difference between the two bone types. In the calvaria, the bone marrow is intertwined with the cortical bone making it thinner than in the femur. Thus, the bone area collected in calvaria is smaller than in femur, and the process is more time-consuming.

### Tissue lysis and RNA extraction

Since the Qiagen RNeasy® Micro Kit is recommended by the LCM device manufacturer for RNA extraction and is commonly used after LCM of brain structures,^18^ we originally used it. However, as mentioned above, no RNA was detectable with the Bioanalyzer**.** Consequently, we hypothesized that the lysis technique did not reach all osteocytes trapped in the matrix. We tested three additional lysis techniques to improve the RNA recovery. Among them, two were used to mechanically break the collagen matrix: ceramic beads (Precellys®) and ultrasounds. The third one was the TRIspin method^16^ consisting in the combination of a lysis step with TRI reagent® and a column-based extraction. RIN values obtained either from entire cryosections or from bone pieces recovered after LCM were then used to assess the impact of these additional lysis techniques on the RNA extraction (Table 2, step 5). The RNA quality after these three additional lysis procedures was good (RIN > 7.0) showing no deleterious effect of the additional lysis techniques. However, none of them enabled the detection of RNA from the bone pieces generated by LCM, suggesting that the quantity obtained was still too low to be detected. Thus, the quality and efficiency of the RNA recovery was assessed by RT-qPCR (see “RT-qPCR after column-based RNA extraction”).

It is known that when a small number of cells is collected, column-based RNA extraction (such as when using Qiagen RNeasy® Micro Kit) decreases the efficiency of RNA recovery.^16^ Therefore, we tried to increase the RNA recovery yield by using RNA precipitation methods. We compared the common Qiagen column-based procedure with two precipitation-based methods. RNA precipitation 1 was the widely used TRI reagent®/chloroform method and RNA precipitation 2 was performed with the MasterPure™ Complete DNA & RNA Purification Kit following the manufacturer instructions. In the latter, the bone pieces were lysed and treated with proteinase K; the proteins were then precipitated, followed by an RNA precipitation with isopropanol, to finish with RNA washes and resuspension. The quality and efficiency of RNA recovery with these three procedures were then assessed by RT-qPCR (see “RT-qPCR after precipitation-based RNA isolation”).

### Osteocyte-specific gene expression analysis

#### RT-qPCR after column-based RNA extraction

The goal of this assay was firstly to validate that we recovered mRNA from osteocytes and, secondly, to discriminate between the two methods of bone sample preparation (Method A vs Method C). To achieve these goals, the totality of RNA extracted from bone pieces with RNeasy® Micro Kit was reverse transcribed and submitted to amplification in two steps: a first step used as “pre-amplification” with 10 cycles and then a qPCR with 45 cycles. Seven genes were analyzed: two reference genes (*Gapdh* and *Eef1a1*) and five genes expected to be expressed in osteocytes (*Gja1/Cx43*, *Phex*, *Pdpn*, *Dmp1*, *Sost*). We compared the two bone sample preparations giving the best RNA qualities: (A) samples decalcified before embedding and freezing and (C) non-decalcified samples with direct snap-freezing (Table 2, step 1). Analysis of Ct (threshold cycles) after pre-amplification followed by qPCR showed that the expressions of the seven analyzed genes were better detected from RNA retrieved after sample preparation (C) (Table 3). Therefore, Method C was selected for the last optimization step: comparison of column and precipitation for RNA isolation.

**Table 3:** Comparison of the impact of the sample preparation on the Ct values obtained for 7 genes after RT-qPCR with pre-amplification.

**Sample preparation** ^**a**^	***Eef1a1***	***Gapdh***	***Gja1***	***Phex***	***Pdpn***	***Dmp1***	***Sost***
Method A	15.5	16.6	19.3	–	20.5	15.8	18.0
Method C	9.7	13.5	15.1	17.3	19.5	14.5	14.0

a After sample preparations with Methods A and C, bone pieces were lysed in RLT/βME solution, followed by RNA extraction with the RNeasy® Micro Kit. Ct values were calculated for seven genes after RT and a qPCR procedure preceded by a 10-cycle pre-amplification step. One experiment per method of sample preparation.

#### RT-qPCR after precipitation-based RNA isolation

Finally, we aimed at avoiding pre-amplification to reduce the risk of bias in the quantification. As the column-based method may reduce the RNA recovery yield for a small number of cells,^16^ we decided to compare a column-based method with RNA precipitation methods. To validate the best procedure of RNA isolation (column vs precipitation 1 or 2), nine bone sections (bone samples prepared with Method C) were microdissected, and the bone pieces were lysed. The lysates were pooled and redistributed in nine equal samples that were treated in triplicates with one of the three procedures of isolation. Six genes were then analyzed: one reference gene (*Sdha*), and five genes expressed in osteocytes (*Gja1*, *Phex*, *Pdpn*, *Dmp1*, *Sost*). The Ct values were lower for the precipitation methods (Table 4) than for the column-based RNA extraction. Although these methods may result in lower purity of RNA than the one obtained with columns, these results suggested that we recovered more RNA from the precipitation methods. RNA precipitation 2 gave the best results in terms of Ct value and reliability.

**Table 4:** Comparison of the impact of the RNA extraction method on the Ct values obtained for 6 genes after RT-qPCR without pre-amplification.

**RNA extraction method** ^**a**^	***Sdha***	***Gja1***	***Phex***	***Pdpn***	***Dmp1***	***Sost***
Column-based RNeasy® Micro kit	29.5 29.9 29.6	34.5 34.3 34.1	34.6 34.9 –	35.8 35.8 –	30.9 31.2 31.3	33.1 35.8 –
Precipitation 1 TRI reagent®/chloroform method	30.8 30.7 30.5	31.9 31.9 31.5	34.0 33.9 33.7	35.0 – 35.0	30.1 30.1 25.5	33.4 32.6 31.8
Precipitation 2 MasterPure™ kit	29.6 29.2 29.7	31.6 31.6 31.0	33.0 33.2 32.1	35.6 35.6 35.6	28.4 28.4 28.2	31.0 30.6 29.9

a After sample preparation by Method C, bone pieces were microdissected, and RNA was isolated by the Trizol/chloroform method (Precipitation 1, triplicate) or by the MasterPure™ Complete DNA & RNA Purification Kit (Precipitation 2, triplicate); in comparison, the standard column-based Qiagen procedure (triplicate) was tested. Ct values were obtained after RT-qPCR without pre-amplification step.

We confirmed that we isolated mRNA from osteocytes, and we obtained quantitative and reliable measurements of the expression of their main marker genes without pre-amplification.

## Conclusion

The goal of this work was to set up a reliable LCM protocol to collect specifically osteocytes from bone and extract their mRNA using a device with a UV laser to both ablate and catapult the bone pieces. Indeed, most of the articles analyzing osteocyte gene expression *in vivo* use bulk bones, meaning that the bone marrow is flushed, the bone surface is scrapped, and eventually, few digestions are performed to clean the bone surface.^31^ This method removes cells from the bone surface but does not allow the study of osteocytes specifically, as bone also contains blood vessels and nerves in the matrix. We chose to optimize LCM for the bone tissue to overcome these limitations.

**Figure 3:**
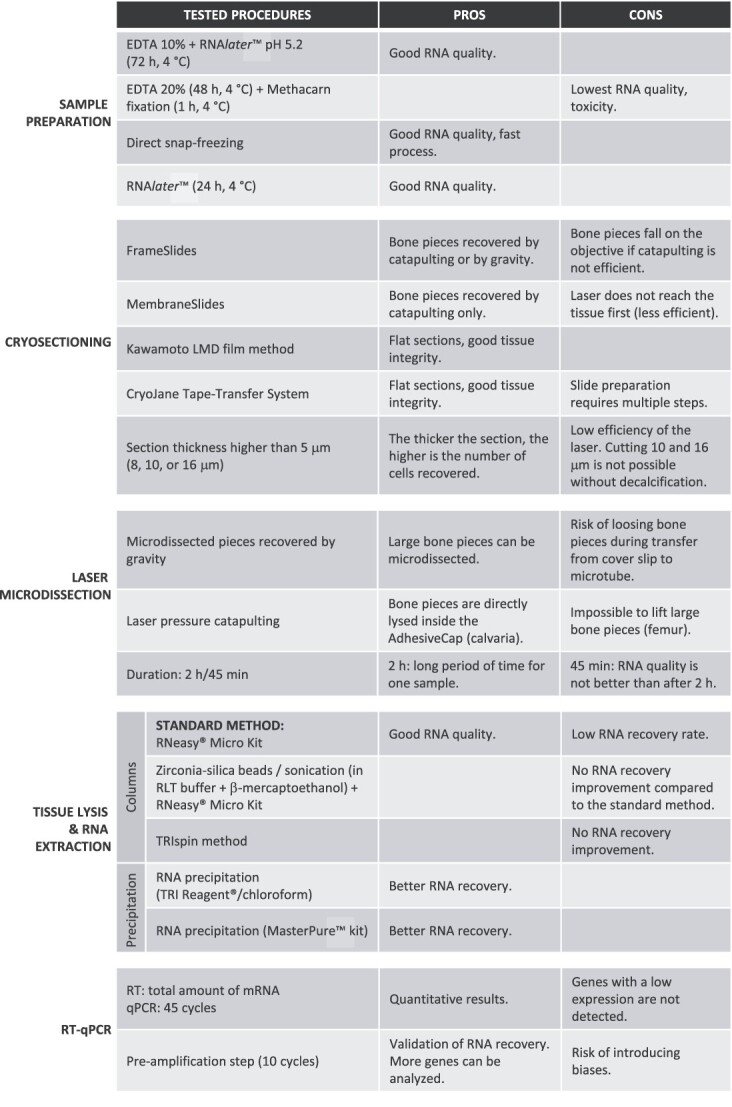
Summary of the methods tested for each step and pros and cons of their utilizations.

The protocol was composed of five steps: sample preparation, cryosection, section treatment, LCM, and lysis/mRNA extraction, which were then followed by RT-qPCR to confirm the presence and quality of mRNA. The main challenges we faced were the poor preservation of tissue section morphology and the risk of RNA degradation during the whole process. Thus, to optimize the procedure of bone microdissection, we explored many alternatives that are reported in Figure 3. For each step, we tried different protocols found in the literature and determined the RIN to evaluate RNA degradation and choose the most suitable protocol. We were able to obtain high-quality RNA from whole bone sections, validating the steps before LCM. However, the RIN of the bone pieces after microdissection was not directly measurable due to the small number of cells collected, leading to a low quantity of RNA recovered. Thus, we evaluated the RIN value thanks to the remaining sections after LCM and considered it similar to the value we would obtain from bone pieces.^24^ By successfully performing RT-qPCR for osteocyte main marker genes, we also confirmed that we isolated RNA from osteocytes.

**Figure 4:**
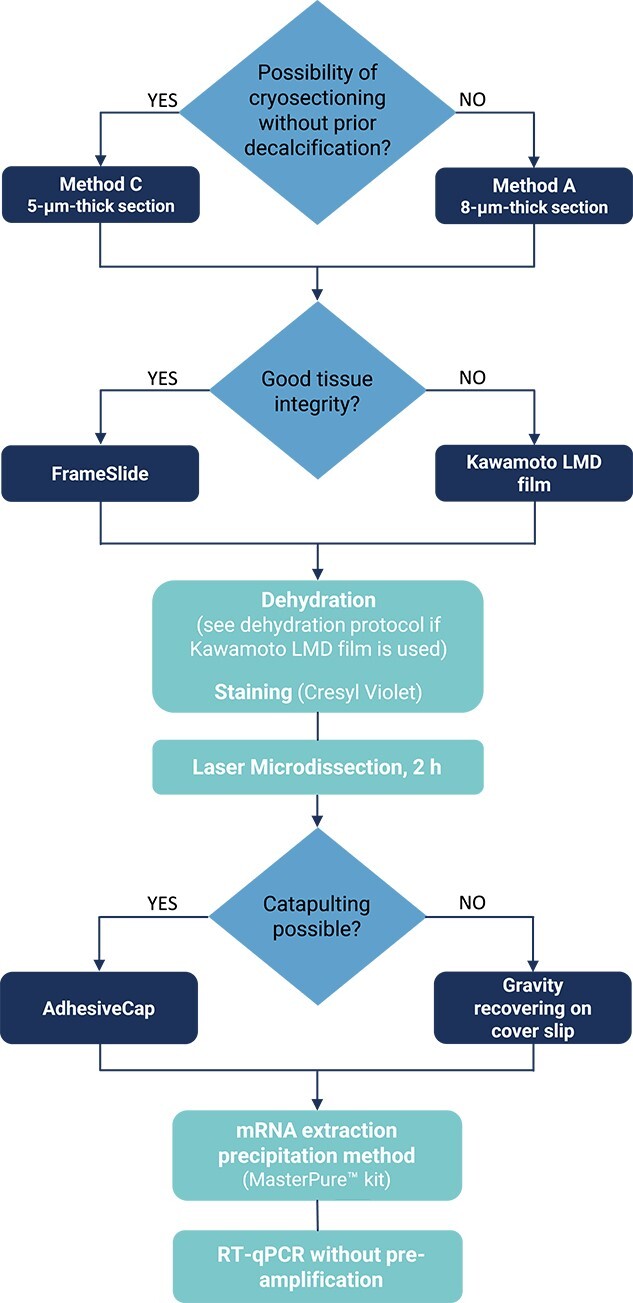
Protocol design flowchart.

We describe in S1 two protocols to extract RNA from osteocytes *in vivo* in mouse femur and calvaria. We also provide a “protocol design flowchart” to guide users working with other bone sample types, other difficult tissues or in other conditions (Figure 4).

Despite the feasibility of LCM for osteocyte gene expression analysis, some limitations remain. LCM is time-consuming (especially when laser cutting cycles must be repeated), and bone cells are dispersed in the matrix, leading to a lower cell number for the same dissected volume than in tissues with a high cell density. Consequently, a small amount of RNA was isolated after 2 h and no more than eight genes could be analyzed with the RT-qPCR technique. To analyze the expression of more genes, we could have performed RNAseq with specific kits for low RNA amounts.

Osteocyte research will benefit from the utilization of different complementary tools. Among them, laser microdissection will help to advance *in vivo* gene expression analysis. As mentioned by bone specialists in recent reviews, there is a real need for a better understanding of osteocyte physiology.^2^^,^^32^ Being able to access osteocytes inside the dense bone matrix will help to decipher their roles in bone physiology and pathology in the future.

## Supplementary Material

Supplementary_information_S1_ziae078

## Data Availability

“The data that supports the findings of this study are available from the corresponding author upon reasonable request.”
