# Supplementary material for: Osteocyte gene expression analysis in mouse bone: optimization of a laser-assisted microdissection protocol
Source: JBMR Plus. 2024 Jun 24;8(8):ziae078. doi: 10.1093/jbmrpl/ziae078 (PMC11264292; doi:10.1093/jbmrpl/ziae078)
Supplement: Supplementary_information_S1_ziae078 [file supplementary_information_s1_ziae078.pdf]

**Supplementary information S1**

**Materials**

| **Product** | **Company** | **Reference/ Catalog number** |
|---|---|---|
| OCT, Optimum Cutting Temperature gel | MM France, Brignais, France | TFM-C |
| SCEM (cryo embedding medium) | SECTION-LAB Co. Ltd., Yokohama, Japan | C-EM001 |
| Molds | MM France | F/E115 |
| Blades | MM France | F/MM35P |
| Kawamoto LMD film | SECTION-LAB Co. Ltd. | C-FL002 |
| Plastic frame | SECTION-LAB Co. Ltd. | C-PS001 |
| Cryofilm fitting tool | SECTION-LAB Co. Ltd. | C-FT000 |
| RNase AWAY™ | Fisher Scientific, Illkirch, France | 11580095 |
| PET FrameSlide | UV technology, Carl Zeiss, Jena, Germany | 415190-9101-000 |
| Cresyl Violet acetate | Sigma-Aldrich Co., St. Louis, MO, USA | C5042 |
| coverslip | 0.13-0.16 mm, Fisher Scientific | 11727055 |
| AdhesiveCap | 500 µL, Carl Zeiss | 415190-9201-000 |
| MasterPure Complete DNA & RNA Purification Kit | Lucigen, Teddington, UK | LU-MC85200 |

**Methods**

Step-by-step protocol for femur (protocol 1) and calvaria (protocol 2).

**Protocol 1 (femur)**

**STEP 1: Bone sample preparation**

The protocol is designed based on experiments with adult mouse bones. The main goal of this step is to prepare the bone samples to allow for efficient cryosectioning and to preserve the RNA integrity.

1. Prepare molds for cryopreservation (1 mold/femur): add OCT (Optimal Cutting Temperature gel, TFM-C); avoid to introduce air bubbles. Let the molds at room temperature.
2. Prepare a container with liquid nitrogen.
3. Euthanize mice by cervical dislocation and immediately dissect the femurs. There is no need to remove the periosteum or flush the bone marrow.
4. Embed bones in OCT and immediately dive the molds in liquid nitrogen.
5. When all the dissections are done, transfer the molds at -80 °C for storage.

**STEP 2: Cryosectioning**

6. Adjust the cryostat temperature to -27 °C ± 1 °C and install a blade specifically designed for hard tissue. Clean all the instruments with RNase AWAY™.
7. When the temperature is reached in the cryostat chamber, place the molds containing the bone samples in the chamber and let them equilibrate in temperature for 15 min. Position one sample on the tissue holder with OCT.
8. Adjust the tissue thickness to 5 µm and transfer the section to a PET FrameSlide; if possible, put 3 sections on the same FrameSlide. The sections should be as flat as

possible to allow the laser beam to cut efficiently. Keep the slide in the cryostat chamber or continue to step 3.

**STEP 3: Dehydration and staining of sections**

9. Prepare containers with ice-cold ethanol 50% (x2), 75% (x2), 95% (x2), and 100% (x2).
10. Dive the FrameSlide successively in ethanol 95%, ethanol 75%, and ethanol 50% for 40 s in each bath.
11. Put the FrameSlide on a paper and add a drop of the solution of Cresyl Violet (1% in ethanol 50%). Let stain for 10 s.
12. Transfer immediately the FrameSlide in the bath of ethanol 50 %, and proceed to dehydration with the successive baths of ethanol 75%, and twice 100% for 40 s in each bath.
13. Let the FrameSlide dry completely at room temperature.

**STEP 4: Microdissection and collection of osteocyte-containing bone pieces**

14. Immobilize a coverslip (0.13-0.16 mm) below the FrameSlide with a tape. The bone section should be between the PET membrane and the coverslip.
15. Place this assembly on the slide holder of the P.A.L.M MicroBeam device with the coverslip below.
16. Adjust parameters depending on the section quality: objective 20x; laser beam energy: 78%; focus: 75%; speed: from 10% to 30%.
17. Draw on the screen the areas that will be microdissected. We recommend to draw and dissect the areas one by one to adjust the number of cutting cycles if necessary.
18. The dissected areas will fall on the coverslip. It is possible to focus on the coverslip to confirm the presence of the bone pieces that were cut.

19. Within 2 hours a total surface of 2.5 ± 0.1 $mm^2$ can be dissected. We have not tested the RNA quality with a longer period of time.
20. After 2 hours, take carefully the FrameSlide, remove the tape that immobilizes the coverslip and add lysis solution; use this solution to transfer the bone pieces into a microtube.
21. Store the microtube containing the bone pieces at -80 °C until RNA extraction.

**STEP 5: Tissue lysis and RNA extraction**

22. Use the MasterPure Complete DNA & RNA Purification Kit, and follow the protocol recommended for tissue samples.
23. Add 1 µL of Proteinase K to the Tissue and Cell Lysis Solution. Homogenize the bone pieces in this solution.
24. Incubate at 65 °C for 15 min, and regularly homogenize by vortexing after 5 and 10 min. Let the solution a few minutes on ice.
25. Add 150 µL of the solution “MPC Protein Precipitation Reagent”, and vortex for 10 s.
26. Centrifuge for 10 min at 4 °C at ≥ 10,000 g to discard the debris. Transfer the supernatant into a new microtube.
27. Add 500 µL of isopropanol. Invert the microtube 30-40 times.
28. Centrifuge for 10 min at 4 °C at ≥ 10,000 g to pellet the nucleic acids.
29. Remove isopropanol without touching the nucleic acid pellet.
30. Rinse with 70% ethanol and centrifuge. Remove all of the residual ethanol. Repeat this step.
31. Resuspend the total nucleic acids in 15 µL of TE Buffer. Store at -80°C until the reverse transcriptase step.

**Protocol 2 (calvaria)**

**STEP 1: Bone sample preparation**

The protocol is designed based on experiments with adult mouse bones. The main goal of this step is to prepare the bone samples to allow for efficient cryosectioning and to preserve the RNA integrity.

1. Prepare molds for cryopreservation (1 mold/calvaria): add SCEM embedding medium (SECTION-LAB Co. Ltd.); avoid to introduce air bubbles. Let the molds at room temperature.
2. Prepare a container of isopentane placed in liquid nitrogen.
3. Euthanize mice by cervical dislocation and immediately dissect the calvaria. Depending on the region of interest (ROI) in the calvaria, it may be interesting to cut the calvaria in the middle or near the ROI (with a scalpel blade) to present this region on an edge of the mold and to cut directly in the ROI after only a few sections.
4. Embed bones in SCEM embedding medium and immediately dive the molds in liquid nitrogen.
5. When all the dissections are done, transfer the molds at -80 °C for storage.

**STEP 2: Cryosectioning**

6. Adjust the cryostat temperature to -27 °C ± 1 °C and install a blade specifically designed for hard tissue. Clean all the instruments with RNase AWAY™.
7. When the temperature is reached in the cryostat chamber, place the molds containing the bone samples in the chamber and let them equilibrate in temperature for 15 min. Position one sample on the tissue holder with SCEM.
8. Cut a few pieces of Kawamoto LMD film with scissors. The adhering part is in the center, framed by two non-adhering regions.

9. Adjust the tissue thickness to 5 µm. Remove the protective membrane from the Kawamoto LMD film and put it in contact with the tissue block; use the cryofilm fitting tool to make the tissue tightly adhere to the film. Perform sectioning. Prepare a few sections (one for each tape piece).
10. Place the film onto the plastic frame provided by the manufacturer.
11. Keep the slide with the films in the cryostat chamber or continue to step 3.

**STEP 3: Dehydration and staining of sections**

12. Prepare containers with ice-cold ethanol 50% (x1), 75% (x1), 95% (x1), and 100% (x2).
13. Put the slide with the films on a paper and add a drop of the solution of Cresyl Violet (1% in ethanol 50 %). Let stain for 10 s.
14. Transfer immediately the slide in the bath of ethanol 50%, and proceed to dehydration with the successive baths of ethanol for 30 s in each bath.
15. Let the slide dry completely at room temperature.

**STEP 4: Microdissection and collection of osteocyte-containing bone pieces**

16. Put the slide on the slide holder of the P.A.L.M MicroBeam device. The side with the bone section should be below, facing the objective, so that the laser beam first cuts the tissue.
17. Adjust parameters depending on the section quality: objective 20x; laser beam energy: 78%; focus: 71%; speed: 30%. Use the option “joint cut” to prevent the bone piece from falling. Apply 3 laser pulses on the bridge and 2 on the bone piece, LPC energy: 80%, focus: 68 %.
18. Put a microtube with the AdhesiveCap just above the slide.

19. Draw on the screen the areas that will be microdissected. A few areas can be determined before starting the laser beam. It is possible to focus on the cap above the slide to confirm that the tissue pieces were catapulted.
20. Within 2 hours, a total surface of 1.5 ± 0.1 $mm^2$ can be microdissected. We have not tested the RNA quality with a longer period of time.
21. After 2 hours, carefully take the microtube, add the lysis buffer and close the cap.
22. Store the microtube containing the bone pieces at -80 °C until RNA extraction.

**STEP 5: Tissue lysis and RNA extraction**

23. Use the MasterPure Complete DNA & RNA Purification Kit, and follow the protocol recommended for tissue samples.
24. Add 1 µL of Proteinase K to the Tissue and Cell Lysis Solution. Homogenize the bone pieces in this solution.
25. Incubate at 65 °C for 15 min, and regularly homogenize by vortexing after 5 and 10 min. Let the solution a few minutes on ice.
26. Add 150 µL of the solution "MPC Protein Precipitation Reagent", and vortex for 10 s.
27. Centrifuge for 10 min at 4 °C at ≥ 10,000 g to discard the debris. Transfer the supernatant into a new microtube.
28. Add 500 µL of isopropanol. Invert the microtube 30-40 times.
29. Centrifuge for 10 min at 4 °C at ≥ 10,000 g to pellet the nucleic acids.
30. Remove isopropanol without touching the nucleic acid pellet.
31. Rinse with 70% ethanol and centrifuge. Remove all of the residual ethanol. Repeat this step.
32. Resuspend the total nucleic acids in 15 µL of TE Buffer. Store at -80 °C until the reverse transcriptase step.
